# Development of individual identification method using thoracic vertebral features as biometric fingerprints

**DOI:** 10.1038/s41598-022-20748-w

**Published:** 2022-09-29

**Authors:** Mitsuru Sato, Yohan Kondo, Masashi Okamoto, Naoya Takahashi

**Affiliations:** 1 Department of Radiological Technology, School of Health Sciences, Faculty of Medicine, Niigata University, 2-746 Asahimachi-dori, Chuo-ku, Niigata, Niigata 951-8518 Japan; 2 Division of Radiological Technology, Graduate School of Health Sciences, Niigata University, 2-746 Asahimachi-dori, Chuo-ku, Niigata, Niigata Japan

**Keywords:** Medical imaging, Medical research

## Abstract

Identification of individuals is performed when a corpse is found after a natural disaster, incident, or accident. DNA and dental records are frequently used as biometric fingerprints; however, identification may be difficult in some cases due to decomposition or damage to the corpse. The present study aimed to develop an individual identification method using thoracic vertebral features as a biological fingerprint. In this method, the shortest diameter in height, width, and depth of the thoracic vertebrae in the postmortem image and a control antemortem were recorded and a database was compiled using this information. The Euclidean distance or the modified Hausdorff distance was calculated as the distance between two points on the three-dimensional feature space of these measurement data. The thoracic vertebrae T1-12 were measured and the pair with the smallest distance was considered to be from the same person. The accuracy of this method for identifying individuals was evaluated by matching images of 82 cases from a total of 702 antemortem images and showed a hit ratio of 100%. Therefore, this method may be used to identify individuals with high accuracy.

## Introduction

Individual identification is performed when a corpse is found after a natural disaster, incident, or accident^1^. Several methods of individual identification, such as dental charts^2–4^ and DNA^5,6^, currently exist. Petju et al.^2^ reported that while dental records can be used for individual identification after a large-scale disaster, such as the 2004 tsunami in Thailand, they do not function efficiently when there is insufficient information. Gin et al.^6^ reported the effectiveness of using DNA identification following the 2018 wildfires in California, USA. In some cases, corpses may be difficult to identify after large-scale disasters due to decomposition or loss of body parts. Individual identification using dental records or DNA identification may not be possible since dental records are required from dentists^7,8^, who may not be available immediately after a disaster. Furthermore, corpses may decompose before the matching of dental records can be completed or matching may not be possible. Loss or decomposition of parts of the corpse with important dental information also makes individual identification difficult and may lead to incorrect identification.

Several studies have reported the development of methods of individual identification using radiological images^9–15^. Morishita et al.^9^ described the use of chest X-ray images as biological fingerprints and showed how to apply them to picture archiving and communication systems. Dedouit et al.^11^ reported that “When a body is not identified or is unidentifiable, as with skeletal, charred, putrefied or mutilated individuals, radiological investigation is necessary.” They also reported that storing data as computed tomography (CT) data enabled analysis regardless of the state of preservation of the corpse.

There have been several disasters in many parts of the world, such as the Indian Ocean tsunami disaster in Thailand^2^ and the Tohoku earthquake in Japan^16^. The death counts at the time of these disasters were in the tens of thousands and individual identification was not possible in some cases. Decomposition of the human remains reduces the number of applicable individual identification methods^17^. Individual identification was performed after the Tohoku earthquake in Japan using visual identification and dental X-ray, and 99% (15,736/15,892) of individuals were identified in 4 years^18^. However, 2573 corpses were not identified after 4 years. Visual identification is subjective and may be erroneous. Therefore, the use of CT scanning is important, although Iino et al.^18^ concluded that “Postmortem CT imaging was not a part of this disaster victim identification operation due to several difficult issues. CT images should be able to be used in future disaster victim identification efforts for mass natural disasters.” Although winter prevented the decomposition of the corpses to some extent after this disaster, CT scans would be useful at certain times of the year, depending on when the disaster occurs.

The Nankai megathrust earthquakes that are predicted to occur in Japan in the future^19,20^ are expected to cause extensive damage and large-scale death; therefore, it is urgent to establish a method of individual identification using body parts that are rarely damaged.

As mentioned above, various methods of individual identification have been developed. However, nevertheless, there are still some corpses with no individual identification, which is a problem. Because individual identification of corpses is difficult by conventional methods in many cases due to loss or decomposition of the body, more individual identification methods need to be developed as much as possible. The present study examined the possibility of using the thoracic vertebrae as a biological fingerprint that is less affected by decomposition and easier to obtain when corpses are found. We developed an individual identification method using the thoracic vertebral features (TVFs) as a biological fingerprint. The shortest diameter in height, width, and depth of the thoracic vertebrae in the postmortem image and a control antemortem were recorded and a database was compiled using this information. The Euclidean distance or the modified Hausdorff distance was calculated as the distance between two points on the three-dimensional feature space of these measurement data. We investigated the accuracy of individual identification using this method. As a result, we report that individual identification was found to be possible with high accuracy.

## Results

**Figure 1:**
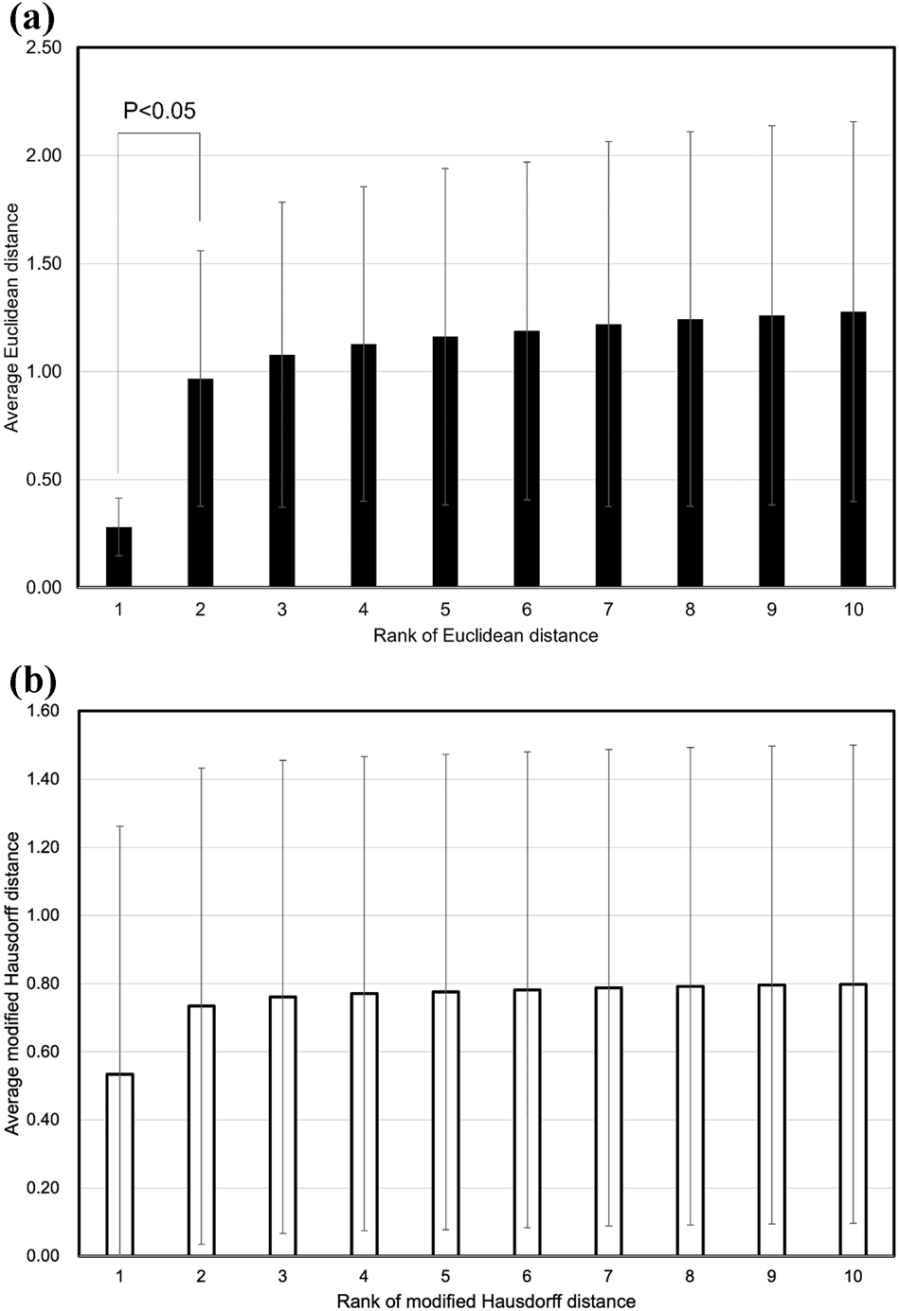
(**a**) Average Euclidean distance for each of rank of Euclidean distance. (**b**) Average modified Hausdorff distance for each of rank of modified Hausdorff distance.

**Figure 2:**
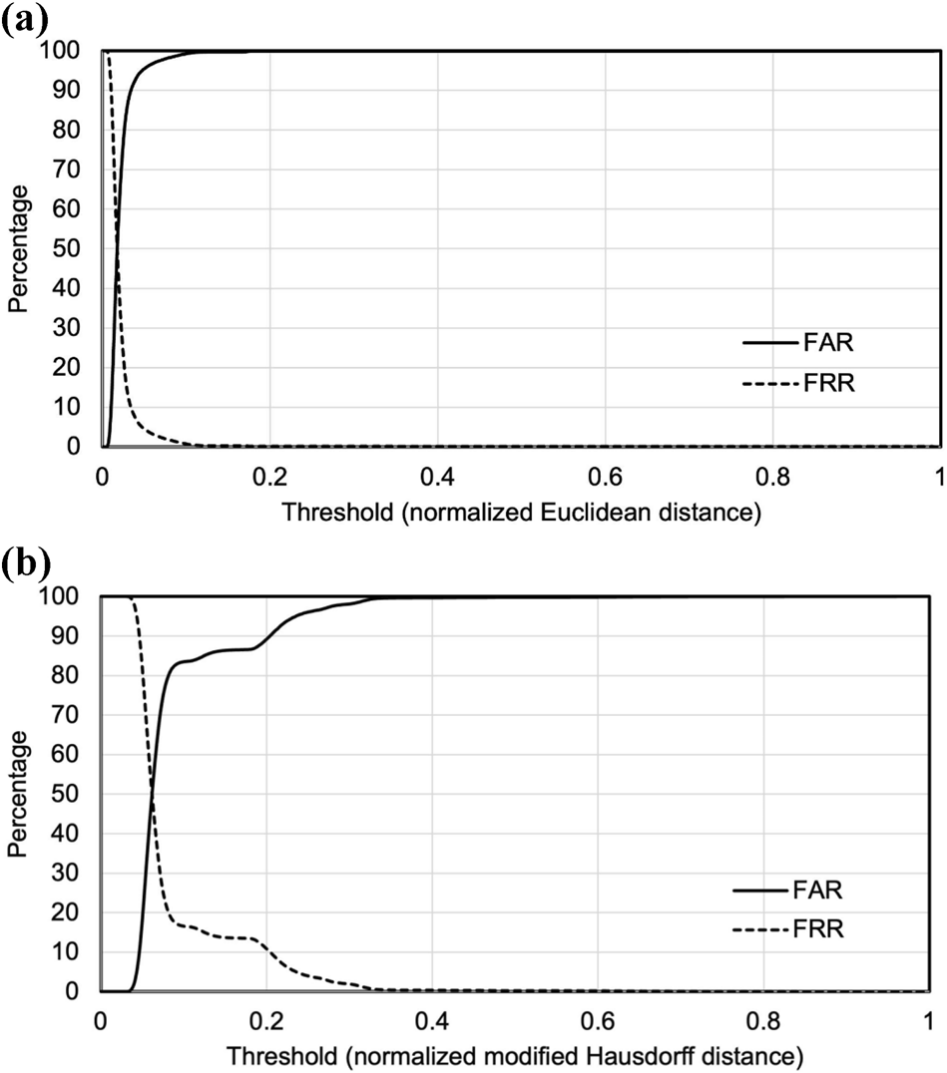
(**a**) FAR and FRR for normalized Euclidean distance. (**b**) FAR and FRR for normalized modified Hausdorff distance.

**Figure 3:**
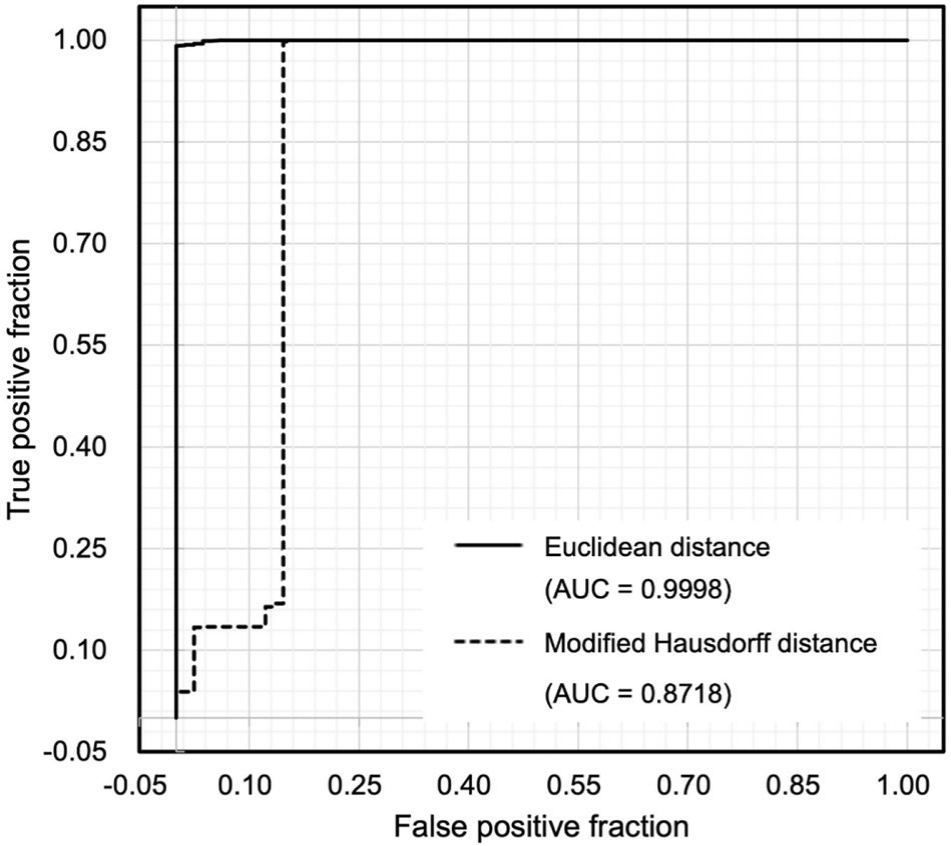
ROC curve of Euclidean distance of modified Hausdorff distance.

The antemortem and postmortem TVF measurement data for all identified cases were in the top 10. In other words, the hit ratio of 100% defined in the present study was achieved (Table 1). In 81 of 82 cases, the Euclidean distance between the antemortem and postmortem TVFs measurement data was the smallest. In one of the 82 cases, the Euclidean distance between the antemortem and postmortem TVF measurement data was the third smallest. In other words, the number of candidates could be reduced to three. The average Euclidean distance for each of rank of Euclidean distance is shown in Fig. 1a. When the magnitude of each Euclidean distance was ranked in decreasing order, the average Euclidean distance between the antemortem and postmortem thoracic vertebrae shape measurement data that ranked first was 0.28 ± 0.13 mm, second was 0.97 ± 0.59 mm, third was 1.08 ± 0.71 mm, fourth was 1.13 ± 0.73 mm, fifth was 1.16 ± 0.78 mm, sixth was 1.19 ± 0.78 mm, seventh was 1.22 ± 0.84 mm, eighth was 1.24 ± 0.87 mm, nineth was 1.26 ± 0.88 mm, and 10th was 1.28 ± 0.88 mm. Student’s *t* test analysis showed that the mean Euclidean distance for the first rank was significantly smaller (*P* < 0.05) than that for the second rank. A false acceptance ratio (FAR) and a false reject ratio (FRR) with a threshold defined for the normalized Euclidean distance are shown in Fig. 2a. An equal error rate (EER) was 50.0 at a threshold of 0.018. the modified Hausdorff distance was also investigated in this study as a comparison. The hit ratio of 100% was achieved (Table 2). In 81 of 82 cases, the modified Hausdorff distance between the antemortem and postmortem TVFs measurement data was the smallest. In one of the 82 cases, the modified Hausdorff distance between the antemortem and postmortem TVF measurement data was the third smallest. The average modified Hausdorff distance for each of rank of modified Hausdorff distance is shown in Fig. 1b. When the magnitude of each modified Hausdorff distance was ranked in decreasing order, the average modified Hausdorff distance between the antemortem and postmortem thoracic vertebrae shape measurement data that ranked first was 0.53 ± 0.73 mm, second was 0.73 ± 0.70 mm, third was 0.76 ± 0.69 mm, fourth was 0.77 ± 0.70 mm, fifth was 0.78 ± 0.70 mm, sixth was 0.78 ± 0.70 mm, seventh was 0.79 ± 0.70 mm, eighth was 0.79 ± 0.70 mm, nineth was 0.80 ± 0.70 mm, and 10th was 0.80 ± 0.70 mm. The FAR and the FRR with a threshold defined for the normalized modified Hausdorff distance are shown in Fig. 2b. The EER was 50.0 at a threshold of 0.062. A receiver operatorating characteristic curve (ROC) is shown in Fig. 3. An Area under the curve (AUC) for individual identification using the Euclidean distance was 0.9998. The AUC for individual identification using the modified Hausdorff distance was 0.8718.

**Table 1:** Rank of Euclidean distance and the Euclidean distance for all cases.

Identification objective	Rank of Euclidean distance	Euclidean distance	Identification objective	Rank of Euclidean distance	Euclidean distance
Corpse 1	1	0.22	Corpse 42	1	0.21
Corpse 2	1	0.18	Corpse 43	1	0.22
Corpse 3	1	0.19	Corpse 44	1	0.19
Corpse 4	1	0.29	Corpse 45	1	0.16
Corpse 5	1	0.19	Corpse 46	1	0.19
Corpse 6	1	0.20	Corpse 47	1	0.26
Corpse 7	1	0.28	Corpse 48	1	0.20
Corpse 8	1	0.31	Corpse 49	1	0.24
Corpse 9	1	0.24	Corpse 50	1	0.22
Corpse 10	1	0.31	Corpse 51	1	0.17
Corpse 11	1	0.19	Corpse 52	1	0.25
Corpse 12	1	0.28	Corpse 53	1	0.19
Corpse 13	1	0.21	Corpse 54	1	0.19
Corpse 14	1	0.38	Corpse 55	1	0.22
Corpse 15	1	0.65	Corpse 56	1	0.22
Corpse 16	1	0.54	Corpse 57	1	0.38
Corpse 17	1	0.29	Corpse 58	1	0.54
Corpse 18	1	0.25	Corpse 59	1	0.42
Corpse 19	1	0.21	Corpse 60	1	0.56
Corpse 20	1	0.25	Corpse 61	1	0.24
Corpse 21	1	0.28	Corpse 62	1	0.38
Corpse 22	1	0.21	Corpse 63	3	0.78
Corpse 23	1	0.28	Corpse 64	1	0.22
Corpse 24	1	0.80	Corpse 65	1	0.20
Corpse 25	1	0.19	Corpse 66	1	0.22
Corpse 26	1	0.15	Corpse 67	1	0.18
Corpse 27	1	0.29	Corpse 68	1	0.24
Corpse 28	1	0.27	Corpse 69	1	0.22
Corpse 29	1	0.20	Corpse 70	1	0.31
Corpse 30	1	0.17	Corpse 71	1	0.25
Corpse 31	1	0.35	Corpse 72	1	0.19
Corpse 32	1	0.35	Corpse 73	1	0.23
Corpse 33	1	0.45	Corpse 74	1	0.22
Corpse 34	1	0.25	Corpse 75	1	0.18
Corpse 35	1	0.33	Corpse 76	1	0.24
Corpse 36	1	0.21	Corpse 77	1	0.42
Corpse 37	1	0.27	Corpse 78	1	0.18
Corpse 38	1	0.21	Corpse 79	1	0.81
Corpse 39	1	0.33	Corpse 80	1	0.27
Corpse 40	1	0.27	Corpse 81	1	0.22
Corpse 41	1	0.14	Corpse 82	1	0.17

**Table 2:** Rank of modified Hausdorff distance and the Euclidean distance for all cases.

Identification objective	Rank of modified Hausdorff distance	Modified Hausdorff distance	Identification objective	Rank of modified Hausdorff distance	Modified Hausdorff distance
Corpse 1	1	0.23	Corpse 42	1	0.24
Corpse 2	1	0.22	Corpse 43	1	0.24
Corpse 3	1	0.22	Corpse 44	1	0.22
Corpse 4	1	0.25	Corpse 45	1	0.20
Corpse 5	1	0.23	Corpse 46	1	0.22
Corpse 6	1	0.23	Corpse 47	1	0.26
Corpse 7	1	0.26	Corpse 48	1	0.23
Corpse 8	1	0.27	Corpse 49	1	0.24
Corpse 9	1	0.25	Corpse 50	1	0.24
Corpse 10	1	0.27	Corpse 51	1	0.21
Corpse 11	1	0.21	Corpse 52	1	0.25
Corpse 12	1	0.24	Corpse 53	1	0.22
Corpse 13	1	0.24	Corpse 54	1	0.22
Corpse 14	1	1.34	Corpse 55	1	0.24
Corpse 15	1	3.16	Corpse 56	1	2.18
Corpse 16	1	3.15	Corpse 57	1	2.20
Corpse 17	1	0.26	Corpse 58	1	2.22
Corpse 18	1	1.18	Corpse 59	1	2.20
Corpse 19	1	0.23	Corpse 60	1	2.22
Corpse 20	1	0.26	Corpse 61	1	2.18
Corpse 21	1	0.26	Corpse 62	1	2.20
Corpse 22	1	0.24	Corpse 63	3	2.24
Corpse 23	1	0.25	Corpse 64	1	0.24
Corpse 24	1	0.43	Corpse 65	1	0.23
Corpse 25	1	0.22	Corpse 66	1	0.24
Corpse 26	1	0.20	Corpse 67	1	0.22
Corpse 27	1	0.27	Corpse 68	1	0.25
Corpse 28	1	0.25	Corpse 69	1	0.24
Corpse 29	1	0.22	Corpse 70	1	0.27
Corpse 30	1	0.21	Corpse 71	1	0.25
Corpse 31	1	0.29	Corpse 72	1	0.23
Corpse 32	1	0.29	Corpse 73	1	0.23
Corpse 33	1	0.34	Corpse 74	1	0.24
Corpse 34	1	0.24	Corpse 75	1	0.23
Corpse 35	1	0.28	Corpse 76	1	0.25
Corpse 36	1	0.24	Corpse 77	1	0.32
Corpse 37	1	0.26	Corpse 78	1	0.22
Corpse 38	1	0.23	Corpse 79	1	0.40
Corpse 39	1	0.27	Corpse 80	1	0.25
Corpse 40	1	0.26	Corpse 81	1	0.23
Corpse 41	1	0.19	Corpse 82	1	0.21

## Discussion

The method developed in the present study can be used for cases where there is a high probability of partial loss of a corpse, such as in large-scale disasters. The use of thoracic vertebrae as a biometric fingerprint method may be effective for the identification of corpses. Furthermore, this method can be used regardless of the number of vertebrae remaining in the corpse. It is possible to automatically recognize the number of remaining vertebrae from the antemortem and thoracic vertebrae measurement data remaining in the corpse and extract and analyze only the data for the existing vertebrae. It is also possible to identify individuals regardless of the number of remaining thoracic vertebrae in the corpse in addition to using thoracic vertebrae that are not easily lost. Therefore, this method can be applied to more cases than the conventional methods using dental records or DNA. In particular, developing a thoracic spine database prior to the Nankai megathrust earthquakes, which are expected to occur in Japan in the future, may help to identify to cases that are difficult to identify quickly using conventional methods.

The contribution of this study is the development of a new method of individual identification with new biological fingerprint. In cases where a corpse is damaged due to a disaster and/or has decomposed over time, the number of usable biological fingerprint of individual identification would be reduced. Therefore, development of new methods of individual identification is needed on an ongoing basis. Recently, Dong et al.^21^ developed an individual identification method using the sphenoid sinus. This study revealed that the three-dimensional shape of the sphenoid sinus can be used as a biological fingerprint. Li et al.^22^ developed the forensic identification method using computer-aided superimposition of the frontal sinus via 3-dimensional reconstruction. In previous study, individual identification methods using 2-dimensional X-ray or CT images existed. This study revealed that the three-dimensional shape of the frontal sinus can also be used as a biological fingerprint. Our method followed these studies and showed that thoracic vertebral features can be used for individual identification. We believe this is an important contribution to the field of forensic radiology.

In the present study, 82 of the 702 cases were individually identified to validate this method. A hit ratio of 100% was achieved in the present study, indicating that individuals can be identified with high accuracy using this method. In 81 of the 82 cases, the Euclidean distance between the antemortem and postmortem TVF measurement data was the smallest. In other words, 98.8% of the predicted results were completely identifiable. In 1 of the 82 cases, the Euclidean distance between the antemortem and postmortem thoracic vertebrae shape measurement data was not the smallest value. However, the Euclidean distance for the same person was the third smallest value, thus reducing the number of candidates to at least three. Furthermore, the values of each Euclidean distance when ranked in order of descending Euclidean distance had the largest difference between the first and second ranks, and a significant difference existed. Therefore, we consider the Euclidean distance to differ significantly between individuals. However, in some cases, such as the only case in the present study in which the Euclidean distance for the same person was not the smallest, the first to third ranks did not differ much (0.62, 0.74, and 0.78). Only three vertebrae were used in this case, which made it difficult to differentiate this case from others. The number of vertebrae to be analyzed should be investigated in the future. The results of individual identification using the modified Hausdorff distance were almost equivalent to the results of individual identification using the Euclidean distance. The cases that failed to be identified were also the same cases (case 63), and their ranking was also the same (third rank). However, as shown in the ROC curve of Fig. 3, the individual identification method using the Euclidean distance performed better than the individual identification method using the modified Hausdorff distance. The AUC was also higher for the Euclidean distance. Furthermore, there was no significant difference between each rank in the modified Hausdorff distance, but there was a significant difference between the first and second ranks in the Euclidean distance. Therefore, we consider that individual identification is more accurate using the Euclidean distance.

We investigated whether individual identification is possible by defining threshold values for Euclidean distance and modified Hausdorff distance using this method as a method of verification. FAR, FRR, and EER for individual identification using Euclidean distance and modified Hausdorff distance were calculated as verification metrics. The results showed that both were almost equivalent, shown in Fig. 2a,b. Decreasing threshold values resulted in smaller FAR and larger FRR. The EER for the individual identification method using Euclidean distance was 50.0 when the threshold value was 0.018. In other words, when the Euclidean distance is less than 0.018, they can be judged to be possibly the same person. The EER for the individual identification method using modified Hausdorff distance was 50.0 when the threshold value was 0.062. In other words, when the modified Hausdorff distance is less than 0.062, they can be judged to be possibly the same person. This investigation indicated a high EER value. However, the accuracy can be ensured even when the number of cases registered in the database is small, at least by defining a threshold for the Euclidean or the modified Hausdorff distance where the FAR is low and the FRR is high, as shown in Fig. 2a,b.

The similarity determination method through the minimum Euclidean distance or the minimum modified Hausdorff distance was used in this study. These methods have limitations in explaining the three-dimensional structure and its variations.

The advantage of this method is its applicability to various cases by obtaining antemortem and postmortem CT data of the thoracic spine. In particular, CT data on the thoracic spine is obtained during screening and it is possible to identify the individuals with high accuracy if a corpse can be CT scanned and the TVFs can be measured. Thus, when a person whose TVFs are not recorded in the database needs to be identified, it is possible to reduce the number of candidates in advance based on individual identification results obtained from dental records and other remaining biological fingerprints to identify the individual with high accuracy by measuring the thoracic vertebrae of the candidates. Conversely, if a database of thoracic vertebrae already exists, the individual can be identified more quickly and accurately if this method is applied first to reduce the number of candidates and identify the individual from dental records and other biological fingerprints. The “hit” defined in the present study refers to the Euclidean distance of the same person's antemortem and postmortem data, which must be at least within the top 10. We believe that this evaluation is suitable because other methods can be used to identify individuals with even higher accuracy and because it enables rapid individual identification by reducing the number of candidates.

The minimum thoracic spine measurement data required to achieve adequate individual identification accuracy remains unclear because evaluation of the hit ratio and number of candidates according to the number of thoracic vertebrae was not performed. Data should be randomly selected for future analysis to investigate the accuracy of individual identification using one thoracic vertebrae compared with 12 thoracic vertebrae, as was performed in the present study. It is important to determine the accuracy of individual identification in relation to the number of thoracic vertebrae used. However, some thoracic vertebrae were out of the imaging range in 12 of the 82 cases used for individual identification in the present study and some TVF measurement data were not present. However, a hit ratio of 100% was achieved in the present study, indicating that highly accurate individual identification could be achieved even using less TVF measurement data.

Although the present study only analyzed the thoracic vertebrae, individual identification may be possible for other vertebrae. Future studies are required to examine whether other vertebral features can also be used as a biological fingerprint by creating a database. The number of applicable cases will increase if identification is possible using any vertebrae. It is also necessary to verify whether individual identification is possible using data obtained from CT scans of the thoracic vertebrae or measurements of the actual thoracic vertebrae in order to verify whether it is possible to use white-boned cadavers.

The time required for this measurement process, which requires measurement of the thoracic vertebrae, needs to be reduced as measurement using a single CT data set requires approximately 20 to 30 min. Therefore, an automatic vertebrae measurement system is required in future.

TVFs can be used as biometric fingerprints. The results of individual identification based on the method in the present study showed that it was possible to identify individuals with a high accuracy. Thus, individuals could be identified quickly if the thoracic vertebrae exist when the corpse is found. In addition, the method developed in the present study could be used to reduce the number of candidates for personal identification. There is potential for further improvement in accuracy using the method used in this study and combining it with other methods to identify individuals according to the state of damage and decomposition of the corpse.

## Methods

### Study overview

**Figure 4:**
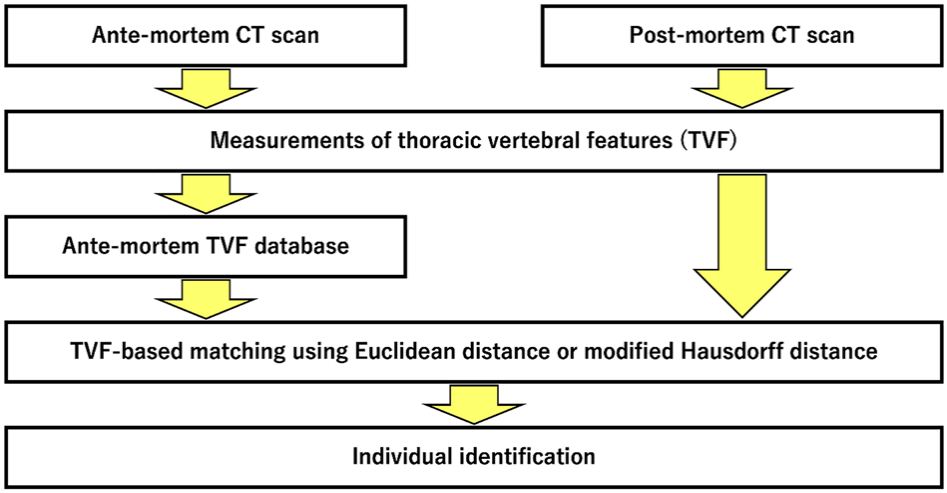
Flowchart of the study method.

The present study aimed to develop an individual identification method using TVFs as biological fingerprints and CT data containing data about antemortem and postmortem TVFs. We used a flowchart of events from finding a corpse to individual identification (Fig. 4) to prepare an antemortem database and corresponding postmortem data to evaluate the accuracy of individual identification.

### CT images

**Figure 5:**
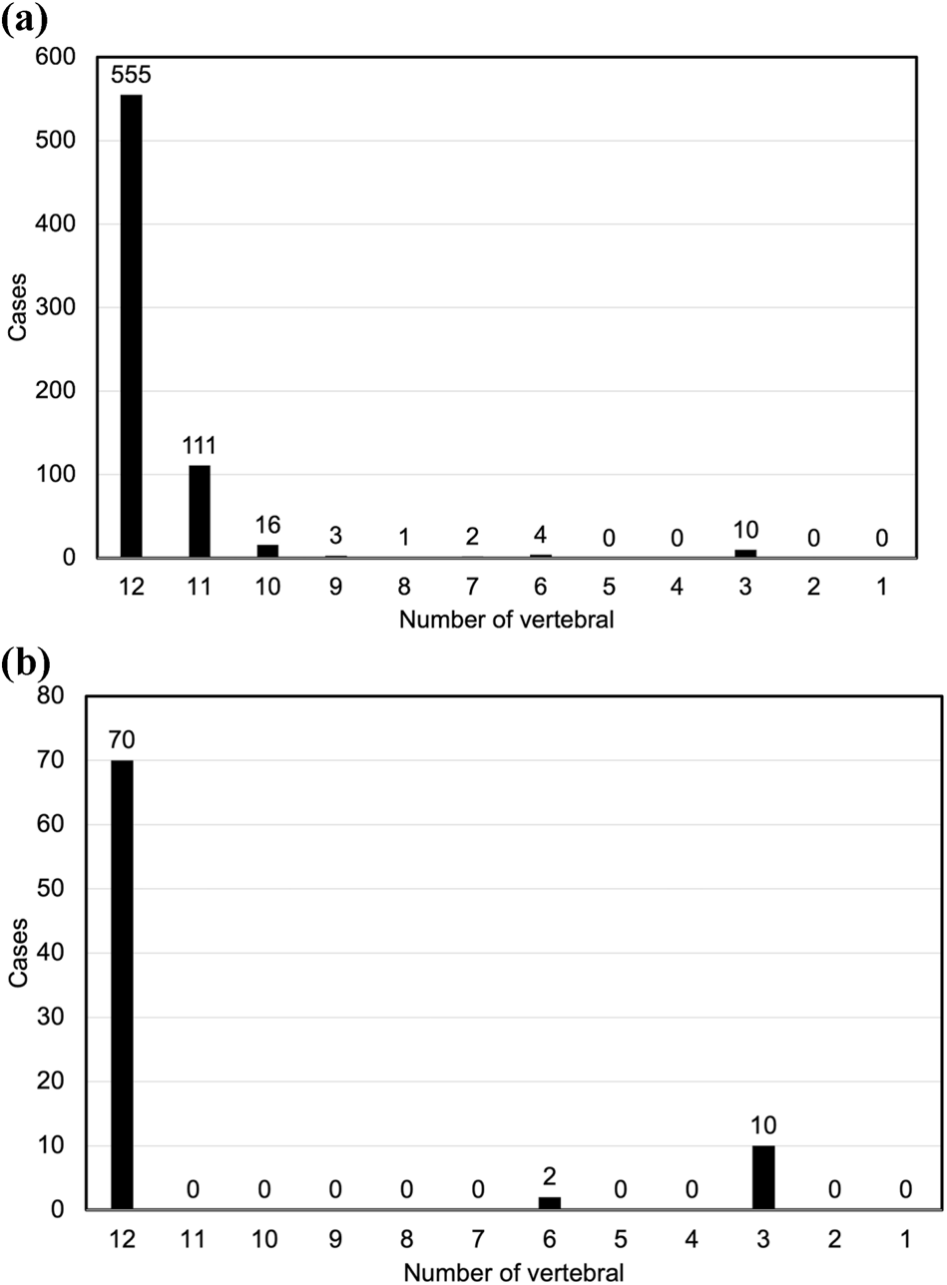
(**a**) Number of thoracic vertebrae existing in the CT data (702 cases; antemortem database). (**b**) Number of thoracic vertebrae existing in the CT data (82 cases of postmortem data).

The prepared CT data included 620 antemortem cases and 82 antemortem and corresponding postmortem cases. The accuracy of this individual identification method was evaluated using TVF-based matching using the Euclidean distances or the modified Hausdorff distance of 82 cases (56 males, 26 females) among a total of 702 antemortem cases. The imaging conditions for these CT data were: tube voltage, 120–140 kV; tube current, 30–725 mA, and slice thickness, 0.6–3 mm. Although CT data included all the thoracic vertebrae, CT data from 147 of the 702 cases did not include some of the thoracic vertebrae (Fig. 5a). The CT data for 12 of the 82 cases for which antemortem and postmortem data existed did not include some thoracic vertebrae data (Fig. 5b). All antemortem data of the 620 cases were obtained from The Lung Image Database Consortium image collection^23^. The 82 cases for which antemortem and postmortem images were obtained were scanned at Niigata City General Hospital (Niigata, Japan) and the Ethics Committee of this hospital approved their use in this study (Acceptance number: 16–003, Decision date: 4/13/2016, Title: Development of an individual identification method based on comparison of antemortem/postmortem X-ray images.). Since these images were obtained from a dead people, the Ethics Committee considered that informed consent was unnecessary. All procedures performed in studies involving human participants were in conducted in accordance with the ethical standards of the institutional and/or national research committee and the 1964 Declaration of Helsinki and its later amendments or comparable ethical standards.

### Measurement method for TVFs

**Figure 6:**
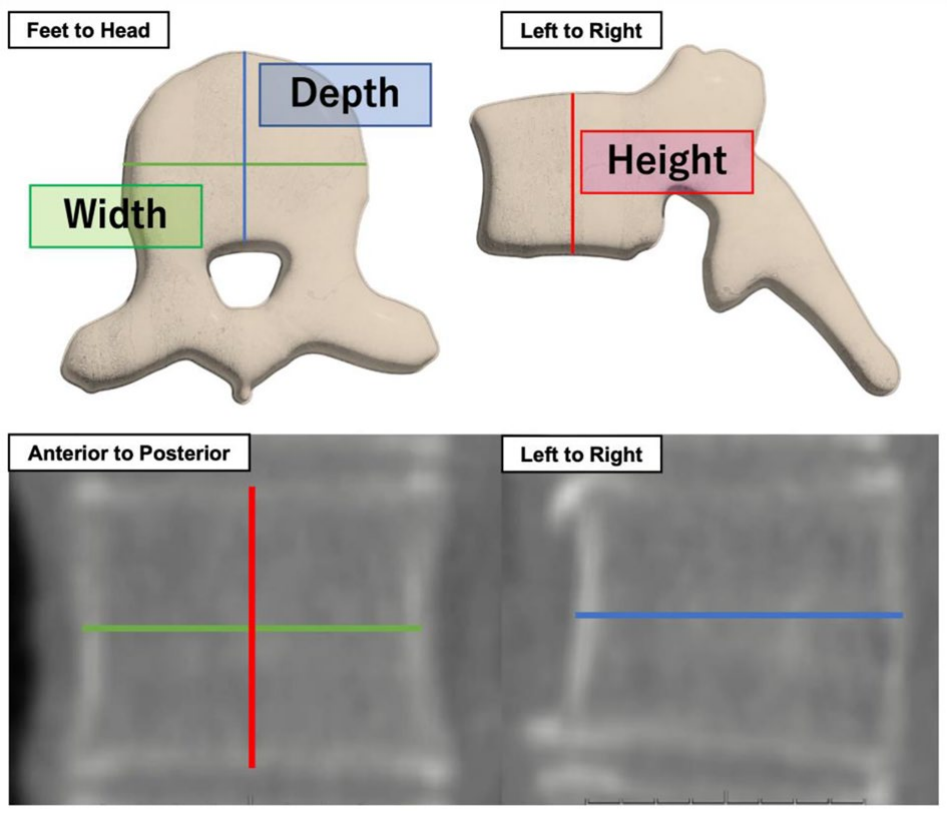
Measurement location of the thoracic vertebrae.

**Figure 7:**
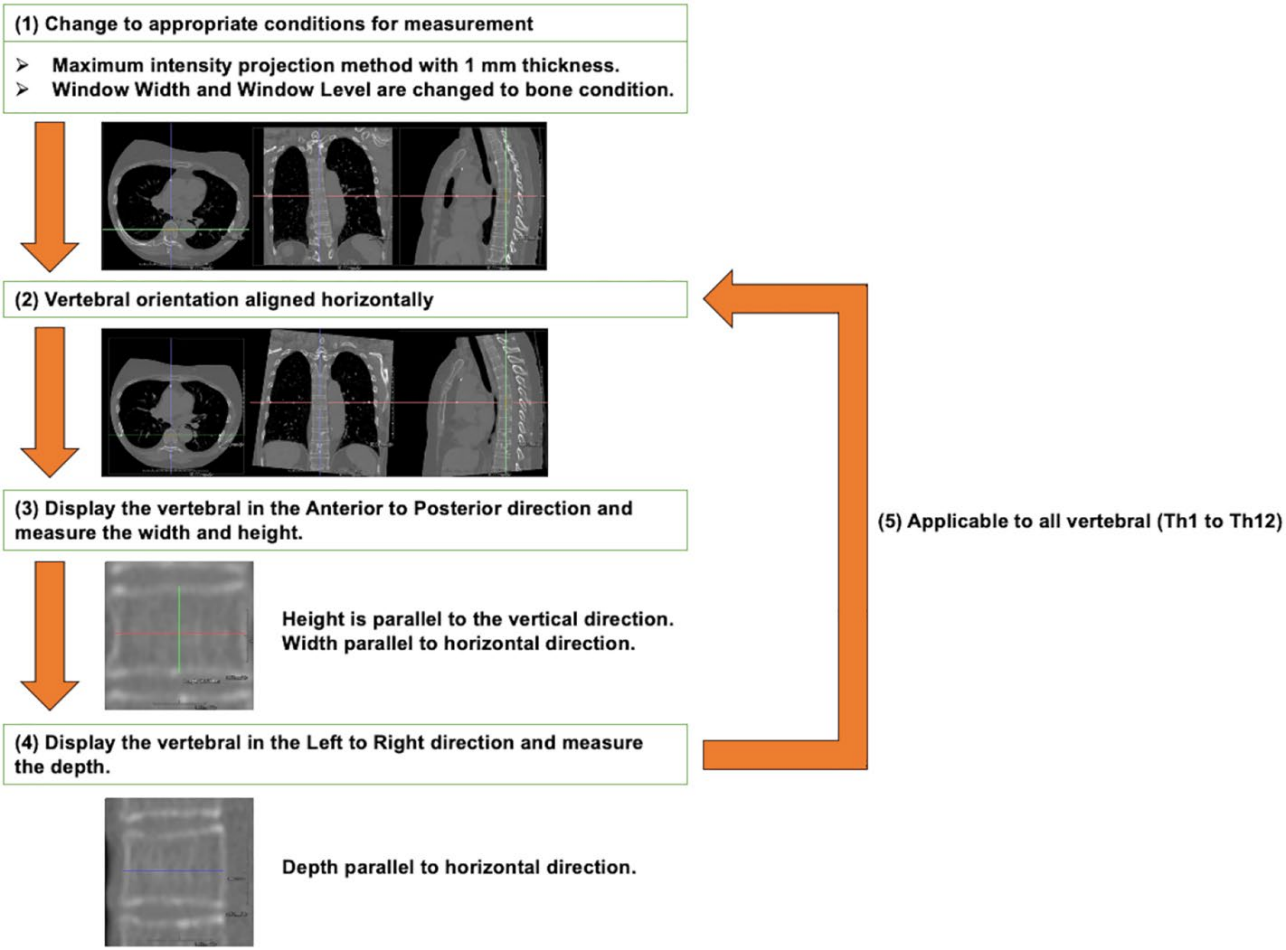
Overview of measurement method for thoracic vertebral features.

The shortest diameter (mm) of the TVF (height, width, and depth) was measured as the biological fingerprint for individual identification (Fig. 6). The width and depth were defined as line segments passing through the center of the height, which was defined as a line segment passing through the center of the width and depth. Aquarius NET (TeraRecon Inc., NC, US) was used as the image display system to measure the TVF. A flowchart of the TVF measurement method is shown in Fig. 7. The TVF measurement method was as follows. (1) The thickness of the image was set to 1 mm using the maximum intensity projection method to facilitate the selection of the most lateral part of the thoracic vertebrae on the CT data. The window width and window level were set to the bone conditions preset in Aquarius NET (window width, 616 and window level, 258). This enabled the contour of the thoracic vertebrae to be clearly visualized. (2) The vertebrae to be measured were oriented so that they are not tilted with the horizontal direction on the image. It was necessary to orient each vertebrae because the thoracic vertebrae were oriented differently along the physiological curvature. (3) The vertebrae were displayed in an anterior to posterior direction and the width and height were measured. Measurement of the width was made parallel to the horizontal direction. Measurement of height was made in parallel to the vertical direction. (4) The left to right direction was displayed and the depth was measured. Measurement of the depth was made parallel to the horizontal direction. (5) TVF measurements were performed on all thoracic vertebrae that could be measured.

### Method for individual identification using the Euclidean distance or the modified Hausdorff distance

The shortest diameters in height, width, and depth of the thoracic vertebrae of the corpse (postmortem data) obtained using the TVF measurement method were recorded. The shortest diameters in height, width, and depth of the thoracic vertebrae of the antemortem data obtained from the same TVF measurement method were recorded and an antemortem database was compiled. The Euclidean distance or the modified Hausdorff distance was calculated as the distance between two points in the three-dimensional feature space at the TVF (height, width, and depth) of the antemortem and postmortem data. The antemortem database (set of antemortem data) was termed U. The distance *TH*_*i*_ (p_j_ ∈ U,q) between the “j”th recorded antemortem data, p_j_, and postmortem data, q, was expressed using formula. The equation of this method using the Euclidean distance is as follows (1):

1 $${TH}_{i}\left({p}_{j}\in U,q\right)=\sqrt{{\left({q}_{H}-{p}_{jH}\right)}^{2}+{\left({q}_{W}-{p}_{jW}\right)}^{2}+{\left({q}_{D}-{p}_{jD}\right)}^{2}}$$

where *p*_*jH*_ represents the measured height of the antemortem data, *p*_*jW*_ represents the measured width of the antemortem data, and *p*_*jD*_ represents the measured depth of the antemortem data. Similarly, *q*_*H*_, *q*_*W*_, and *q*_*D*_ represent the height, width, and depth measurements of the postmortem data, respectively. The equation of this method using the Hausdorff distance is as follows (2):

2 $${TH}_{i}\left({p}_{j}\in U,q\right)=max\left\{min\left\{distance\left(\left[{p}_{jH}, {p}_{jW},{p}_{jD}\right],\left[{q}_{H}, {q}_{W}, {q}_{D}\right]\right)\right\}\right\}$$

In this study, a modified Hausdorff distance is used, which is an improvement of the Hausdorff distance^24^. *TH*_*i*_(p_j_ ∈ U,q) represents the “n”th data of the measured thoracic vertebrae in the “j”th recorded antemortem data, p, and postmortem data, q, where the maximum number of thoracic vertebrae (12) is the maximum value of n. Thus, the total Euclidean distance or the modified Hausdorff distance *SUMTH*_*i*_(p_j_ ∈ U,q) when the number of measured thoracic vertebrae in the “j”th recorded antemortem data is n can be expressed as follows:

3 $${SUMTH}_{i}\left({p}_{j}\in U,q\right)= \sum_{i=1}^{n}{TH}_{i}({p}_{j}\in U,q)$$

Thus, the set with the smallest value among all *SUMTH*_*i*_(p_j_ ∈ U,q) is considered to be the same person. However, the smaller the number of the vertebrae used in the calculation of the Euclidean distance or the modified Hausdorff distance, the smaller the total Euclidean distance or the modified Hausdorff distance. Therefore, individual identification may not be able to be performed correctly for some case due to the number of vertebrae remaining in the corpse. Therefore, we divided *SUMTH*_*i*_(p_j_ ∈ U,q) by the number of measured vertebrae n in the “j”th recorded antemortem data to enable individual identification regardless of the number of vertebrae that were available for measurement as follows:

4 $$E(m)={Asc.Rank}_{m}\left[\frac{{SUMTH}_{i}\left({p}_{j}\in U,q\right)}{n}\right]$$

where E is the set of the Euclidean distances or the modified Hausdorff distance between the “j”th recorded antemortem data, p_j_, and postmortem data, q, averaged over the number of vertebrae used in the analysis. Asc.Rank_m_ represents a function that reorders the set U of antemortem data, p_j_, in Euclidean distance order (ascending order) between antemortem data, p_j_, and postmortem data, q. Thus, E(m) represents the antemortem data, p_j_, with the “m”th smallest Euclidean distance or the modified Hausdorff distance. In other words, this function can sort the candidates for the same person in order of similarity of biological fingerprints to the postmortem data, q. In the present study, m was defined as a value between 1 and 10, and 10 candidates for individual identification were selected.

### Evaluation of the method

A hit ratio was defined to evaluate our identification method. Cases in which the magnitude of the Euclidean distance between the antemortem and postmortem data for the same person were within the top 10 in ascending order were considered a “hit” and represented the percentage of cases hit among the 82 cases with postmortem data. The reason for considering cases where the magnitude of the Euclidean distance was within 10 in ascending order as the hit ratio was to ensure individual identification by combining this method with other methods if the number of candidates could be reduced to at least 10 using this method. In addition, each Euclidean distance was ranked in ascending order and the average value up to the 10th rank was investigated. The average Euclidean distance between the neighboring ranks was also examined for significant differences using student’s *t* test. Statistical analysis was performed using R version 4.2.0 [R Core Team (2022)], which is a language and environment for statistical computing (R Foundation for Statistical Computing, Vienna, Austria. URL https://www.R-project.org/).

In addition, a threshold was defined for the Euclidean distances or the modified Hausdorff distance in this study. Cases that were below the threshold were estimated to be the same person. In other words, we investigated whether the individual identification method using the Euclidean distance or the modified Hausdorff distance could be used as a one-to-one verification method. The FAR, FRR, and EER were calculated in this investigation. The Euclidean distance and the modified Hausdorff distance were normalized to compare individual identification methods. True positive fraction and false positive fraction were calculated from all the Euclidean distance and the modified Hausdorff distance and individual identification results. The area of the ROC curve is called the AUC. If the AUC is large, identification methods are considered to have superior individual identification ability.

### Ethics approval and consent to participate

This work has not been published before in part or its entirety. All procedures performed in studies involving human participants were in conducted in accordance with the ethical standards of the institutional and/or national research committee and the 1964 Declaration of Helsinki and its later amendments or comparable ethical standards.

## Data Availability

The code generated during the current study is available from the corresponding author on reasonable request. However, the image datasets presented in this study are not publicly available due to ethical reasons.
